# Immune-derived cardiac autonomic signatures: predicting autonomic responses to exercise from B-cell phenotypes

**DOI:** 10.3389/fnins.2025.1702281

**Published:** 2026-01-15

**Authors:** Matías Castillo-Aguilar, Ginés Viscor, Lindybeth Sarmiento, Julieta Sepúlveda, Marcelo Navarrete, Cristian Núñez-Espinosa

**Affiliations:** 1 Centro Asistencial Docente y de Investigación (CADI-UMAG), Punta Arenas, Chile; 2 Escuela de Medicina, Universidad de Magallanes (UMAG), Punta Arenas, Chile; 3 Physiology Section, Department of Cell Biology, Physiology and Immunology, Faculty of Biology, Universitat de Barcelona, Barcelona, Spain; 4 Facultad de Ciencias de la Salud, Universidad de Magallanes (UMAG), Punta Arenas, Chile

**Keywords:** aging, autonomic nervous system, B cells, exercise, heart rate variability, immunosenescence

## Abstract

**Objective:**

Aging affects both immune and autonomic regulation, yet their interaction remains poorly characterized. This study investigated how aging B-cell subpopulations, defined by CD21/CD11c expression, are associated with autonomic nervous system (ANS) dynamics, as measured by heart rate variability (HRV) during exercise in older adults.

**Materials and methods:**

In this cross-sectional study, 81 community-dwelling older adults (mean age 70.7 ± 5.8 years) underwent immune flow cytometry profiling of total B cells and four CD21/CD11c phenotypes. Continuous R–R interval (RRi) data were recorded at rest, during a standardized Two-Minute Step Test (TMST), and over a 5-min recovery period. A coupled-logistic RRi-vs-time model capturing each participant’s cardiac autonomic signature (CAS) was obtained. Individual parameter estimates were regressed on standardized immune predictors using multivariate Bayesian models adjusted for age, sex and body composition.

**Results:**

Higher counts of CD21^+^CD11c^+^ B cells were associated with elevated baseline RRi (resting vagal tone), an increased exercise-induced RRi drop, and an incomplete post-exercise recovery. Conversely, greater CD21^−^CD11c^−^ B-cell counts were associated with lower resting RRi, a faster sympathetic-driven RRi decrease during exercise, and more complete vagal reactivation during recovery. High posterior probability (>90%) was observed for the aforementioned posterior estimates.

**Conclusion:**

CD21^+^CD11c^+^ and CD21^−^CD11c^−^ aging B-cell subsets display opposite associations with ANS responsiveness to acute exercise, suggesting immunosenescence-linked autonomic modulation on the neuro-immune axis. Distinct B-cell phenotypes may serve as biomarkers of resilience or fragility in aging, supporting personalized interventions to optimize cardiovascular health in aging individuals.

## 1 Introduction

The intricate interplay between the nervous and immune systems, commonly referred to as the neuro-immunological axis, is fundamental for maintaining physiological homeostasis, ensuring effective responses to stressors and regulating immune responses (Bonaz et al., 2017; Drury et al., 2019; Laborde et al., 2022). The autonomic nervous system (ANS), composed of sympathetic (SNS) and parasympathetic (PNS) branches, mediates bidirectional communication with the immune system. Immune derived cytokines can influence neurotransmitter release, synaptic plasticity and endocrine activity, while autonomic pathways modulate immune cell trafficking and activation (Porter and O’Connor, 2022; Elia and Fossati, 2023).

This crosstalk is essential for acute responses to infection and injury, yet aging disrupts both systems. Immunosenescence, a hallmark of aging, encompasses widespread impairments in innate and adaptative immunity, including diminished output of naïve T and B cells, reduced vaccine responsiveness, and a chronic low-grade inflammatory state known as “inflammaging” (Jurcau et al., 2024). Persistent inflammatory signaling and impaired regulatory control may place sustained pressure on autonomic circuits. Conversely, autonomic dysfunction may actively shape age-related immune phenotypes (Jiang et al., 2022; Giunta et al., 2024). Aging, also shifts autonomic balance towards sympathetic dominance and reduced vagal tone, weakening the anti-inflammatory potential of parasympathetic signaling (Giunta et al., 2024). Together, these dysruptions may lower physiological resilience and contribute to chronic disease.

Within this framework, B lymphocytes warrant particular attention. In addition to antibody production, B cells function as antigen-presenting cells and cytokine producers, influencing inflammatory and neuroimmune dynamics (Wang et al., 2020; Engler-Chiurazzi, 2024). With aging, the B cell compartment undergoes marked phenotypic shifts, including reduced naïve cells and an expansion of memory and atypical subsets, such as age-associated B cells (ABCs). Immunophenotyping of B cells based on CD21 and CD11c expression allows for the delineation of functionally distinct B cell subsets: CD21^−^CD11c^+^ cells (ABCs), CD21^+^CD11c^−^ (naïve/resting memory), CD21^+^CD11c^+^ (activated memory), and CD21^−^CD11c^−^ (putatively anergic or dysfunctional) (Rincon-Arevalo et al., 2021). CD11c^+^ B cells, often classified as ABCs, display pro-inflammatory transcriptional profiles, including robust expression of T-bet and Blimp-1 (Frasca et al., 2017), and are expanded in contexts such as aging, autoimmunity, and chronic infections (Golinski et al., 2020). Converseky, CD21, a complement receptor typically expressed on follicular B cells, is downregulated in chronically stimulated, anergic, or functionally exhausted B cell subsets, serving as a phenotypic marker of antigen exposure and altered functional state (Del Padre et al., 2020). These immunophenotypic distinctions provide valuable insights into the immune aging and its functional consequences.

Emerging evidence suggests that immune status may influence autonomic tone, and vice versa. Lymphocytes express receptors for neurotransmitters such as norepinephrine and acetylcholine via adrenergic and cholinergic receptors, and can even produce acetylcholine themselves highlighting a non-neuronal cholinergic signaling network within lymphoid tissue (Halder and Lal, 2021; Jacobson et al., 2021). The vagus nerve is central to this communication through the cholinergic anti-inflammatory reflex, which suppresses cytokine release during systemic inflammation (Tracey, 2002; Bonaz et al., 2017). Disruption of this balance is implicated in cardiovascular, metabolic, and neurodegenerative conditions (Giunta et al., 2024). Aging compounds these disruptions by simultaneously reducing parasympathetic tone, promoting sympathetic dominance, and perpetuating systemic inflammation (Barrera-Salas et al., 2017; Frasca et al., 2023; Giunta et al., 2024). This shift reduces the resilience of the neuro-immune axis to respond to physiological stressors.

Physical activity is a powerful modulator of both autonomic and immune function in older adults. Regular exercise improves vagal tone, enhances heart rate variability (HRV), and reduces systemic inflammation (Benarroch, 2020; Laborde et al., 2022; Olivieri et al., 2024). Immunologically, it is associated with a more balanced lymphocyte profile, with higher proportions of naïve T and B cells and fewer senescent subsets (Weyh et al., 2020; Tylutka et al., 2021). HRV analysis during and after submaximal exercise provides a non-invasive probe of autonomic adaptability (Castillo-Aguilar et al., 2023, 2025). Parametric modeling of HRV trajectories allows for individualized assessments of autonomic dynamics, capturing features such as recovery and flexibility (Castillo-Aguilar et al., 2025). This set of parameters conforms an individual’s Cardiac Autonomic Signature (CAS).

Understanding how aging-associated B cell phenotypes influence autonomic dynamics adaptability, could provide new insights into physiological resilience in older adults. We hypothesize that distinct B cell subpopulations, particularly CD21^+^CD11c^+^ and CD21^−^CD11c^−^ phenotypes, are associated with interindividual differences in autonomic response during submaximal exercise. By integrating high-resolution immunophenotyping with dynamic HRV modeling, this study aims to identify immune correlates of autonomic resilience, and to advance our understanding of the bidirectional interplay between immune aging and CAS regulation.

## 2 Materials and methods

### 2.1 Study design

This study employed an observational, correlational, and cross-sectional design.

Prior to any data acquisition, all participants received a comprehensive explanation of the study’s aims, procedures, and potential outcomes.

### 2.2 Setting

This study took place at the Centro Asistencial Docente e Investigacion (CADI-UMAG), the University of Magallanes academic healthcare and research center associated with the University of Magallanes, in Punta Arenas, Chile. To minimize the impact of circadian rhythms on physiological measurements, all assessments were were conducted between 9:00 and 11:00 a.m. in a controlled environment with constant temperature (~20 °C), artificial lighting and quiet private room, to minimize circadian and environmental influences.

### 2.3 Participants

Participants were recruited from the local community through advertisements and outreach. A total of 81 older adults participated in this study, comprising 56 women and 25 men, with a mean age of 70.7 years (SD = 5.8, range 61–89). Inclusion criteria for participation were: (i) being 60 years or older at the time of enrollment; (ii) permanent residency in the Magallanes and Chilean Antarctic region, ensuring a relatively homogeneous population exposed to similar environmental and socioeconomic factors; (iii) achieving a score above 60% on the Karnofsky Performance Status scale, a common measure of functional capacity, indicating sufficient autonomy to complete the study assessments (Schag et al., 1984); and (iv) no prior diagnosis of conditions that could confound autonomic or cardiovascular function, such as diabetic neuropathy, pacemaker implantation, clinical depression, cognitive impairment, motor disability, or dementia.

Exclusion criteria were implemented to minimize factors that could confound autonomic measurements. Individuals were excluded if they: (i) were using beta-blockers during the study, as these medications can significantly alter autonomic and cardiovascular responses; (ii) had consumed any stimulant substances, including caffeine or sympathomimetic medications, within 12 h prior to cardiac assessment; or (iii) had any degree of motor impairment restricting independent movement that could interfere with study procedures. Importantly, no recruited participants met these exclusion criteria.

### 2.4 Procedures

Participants attended a single study visit after a 12-h fast and abstaining from strenuous exercise and alcohol. Upon arrival, they provided informed consent and underwent a brief health screening to confirm eligibility, including blood pressure assessment (SBP < 140 mmHg and DBP < 90 mmHg).

Sociodemographic data, including name, age, sex, and any existing chronic medical conditions, were collected via a structured interview. This information was used to characterize the study sample and identify potential confounding factors.

Following the initial registry, body composition measurements were performed using multi-frequency bioelectrical impedance analysis (BIA) according to standardized procedures. Participants were asked to empty their bladder immediately prior to this assessment.

A 4 mL peripheral venous blood sample was collected in EDTA tubes by a trained nurse. Importantly, this blood draw ocurred prior to any physical exertion or baseline physiological measurements, to avoid any acute exercise-induced immunological alterations. These samples were gently homogenized and kept at room temperature for subsequent immunophenotypic analysis, being processed within 2 h of collection.

Following a minimum 10-min rest period in a seated position, participants were fitted with a Polar H10 heart rate monitor for continuous R–R interval recording and an Omron Hem-7142 monitor for blood pressure measurements. After baseline physiological measurements (HRV segment at rest, BP), participants performed the Two-Minute Step Test (TMST) as an acute physiological stressor. R–R intervals were recorded continuously during the 2-min test and for a 5-min recovery period immediately afterward. Blood pressure was measured again immediately after completion of the TMST to ensure participant safety.

### 2.5 Assessments

#### 2.5.1 Body composition

Body composition, including body mass index (BMI), body fat percentage, lean muscle mass, body water, and bone mass, was assessed using multi-frequency BIA. Measurements were performed using the Tanita BC-558 Iron-man Segmental Body Composition Monitor (Tanita Ironman, Arlington Heights, USA). Participants were measured following standard BIA recommendations (fasting ≥4 h, bladder emptied). They were also asked to empty their bladder before the measurement. Participants stood barefoot on the device’s foot electrodes and held the hand electrodes while wearing light clothing.

#### 2.5.2 Two-minute step test (TMST)

The Two-Minute Step Test (TMST), a standardized protocol from the Senior Fitness Test battery (Rikli and Jones, 1999), was used as an acute, exercise-induced physiological stressor to challenge cardiac autonomic control. While also providing an index of lower body endurance and functional mobility, the TMST’s primary role in this study was to elicit a controlled, submaximal physiological perturbation suitable for assessing dynamic autonomic responses in older adults. Participants were instructed to march in place for 2 min, lifting their knees to a midpoint between the patella and the iliac crest, guided by verbal cues. The total number of steps completed served as a measure of exercise volume and functional performance. A trained kinesiologist counted the steps, and participants received verbal encouragement to maintain consistent effort and proper form throughout the 2-min period. Rest was allowed if needed, with participants encouraged to resume the test as soon as possible.

During the TMST, R–R intervals were continuously recorded using Polar H10 (Polar Electro Oy, Kempele, Finland) heart rate monitors to assess HRV dynamics before, during, and after exercise. Additionally, blood pressure (BP) was measured immediately before and after the test to evaluate safety, physiological adaptations, and recovery using the Omron Hem-7142 monitor.

The TMST was performed after a 10-min rest period, followed by a 5-min recovery period (12 min total). The total number of steps completed in 2 min was recorded as the outcome measure for functional capacity, while the physiological stress induced by this standardized exertion served as the basis for assessing dynamic autonomic responses.

#### 2.5.3 Immune profiling and B cell phenotyping

To characterize B cell subpopulations, including age-associated B cells (ABCs), we used fluorochrome-conjugated monoclonal antibodies (mAbs) specific for human CD45 (Brilliant Violet 570, clone HI30, BioLegend), CD19 (FITC, clone HIB19, BioLegend), CD21 (APC A750, clone Bu32, BioLegend) and CD11c (PB 450, clone 3.9, BioLegend). A viability marker (7-AAD PC5.5, BioLegend) was also included to exclude dead cells. All antibodies were used at manufacturer-recommended concentrations.

Peripheral blood samples were collected in 4 mL EDTA tubes and processed within 2 h of collection. For each assay, 100 μL of whole blood (approximately 1 × 10^6^ cells) were incubated with the antibody cocktail for 30 min at room temperature in the dark. After staining, red blood cells were lysed by adding 125 μL of OptiLyse C (Beckman Coulter), followed by brief mixing and a 10-min incubation in the dark. Cells were then washed with 1 mL of IsoFlow™ buffer (Beckman Coulter) to remove debris and unbound antibodies.

Stained samples were acquired on a Beckman Coulter Model S flow cytometer (Beckman, CA, USA), and analyzed using FlowJo software (Tree Star, OR, USA). A minimum of 100.000 CD19^+^ events were acquired per sample. Fluorescence compensation was performed using single-stained compensation beads (BioLegend). Fluorescence minus one and isotype-matched Ab controls were used to set analysis gates.

Gating strategy included sequential selection of CD45^+^ lymphocytes, exclusion of 7-AAD^+^ dead cells, and identification of CD19^+^ B cells. B cell subsets were defined based on the differential expression of CD21 and CD11c. Age-associated B cells (ABCs) were defined as CD19^+^CD11c^+^CD21^−^. Additional subsets included CD19^+^CD21^+^CD11c^−^ (naïve/central memory B cells), CD19^+^CD21^+^CD11c^+^ (activated memory B cells), and CD19^+^CD21^−^CD11c^−^ (putative anergic or dysfunctional B cells). Subset definitions were based on previously reported phenotypic profiles of human B cells (Pinto et al., 2023; McGrath et al., 2024).

**Figure 1:**
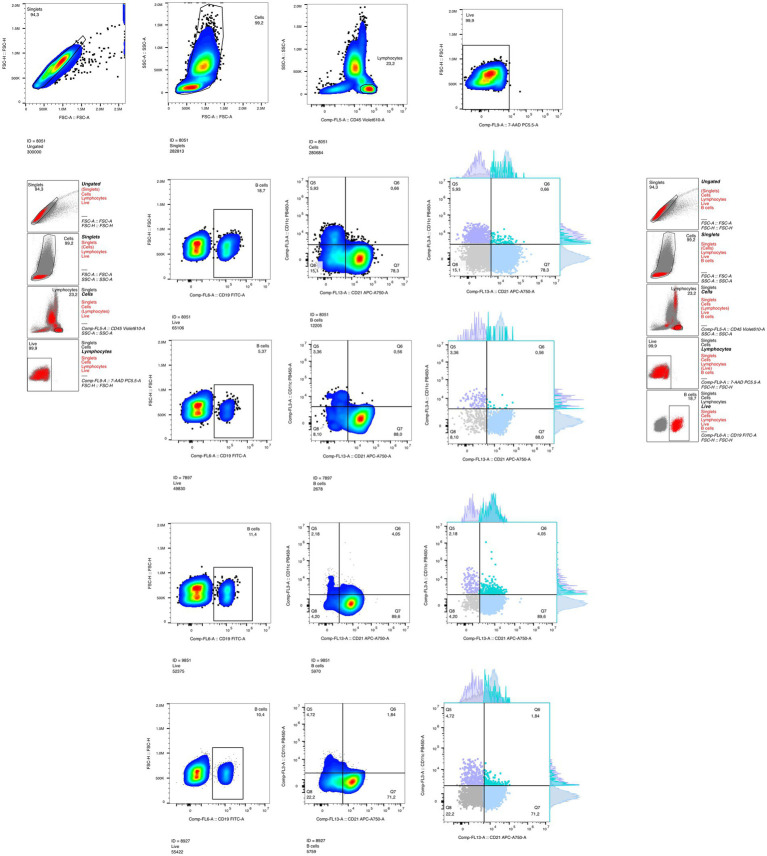
Sequential gating strategy for the identification of ABCs defined as CD19^+^CD11c^+^CD21^−^, naïve/central memory B cells as CD19^+^CD21^+^CD11c^−^, activated memory B cells CD19^+^CD21^+^CD11c^+^, and putative anergic or dysfunctional B cells as CD19^+^CD21^−^CD11c^−^. Four individual samples are shown in the last four rows depicting inter individual variability in CD21/CD11c heterogeneity with each subpopulation highlighted with corresponding marginal distributions, respectively. A minimum of 100,000 CD19^+^ events were acquired per sample.

Sequential gating strategy can be observed in Figure 1.

### 2.6 Statistical analysis

We employed a fully Bayesian modeling framework to characterize how cardiac autonomic modulation responds to exercise in the presence of multiple confounding influences. Bayesian inference was preferred over traditional frequentist methods because it provides complete posterior distributions for all parameters, thereby allowing thorough quantification of uncertainty and probabilistic interpretation via credible intervals (de Van Schoot et al., 2021). By incorporating prior information, either drawn from existing literature or specified as weakly regularizing distributions, we constrained implausible parameter values, mitigated the impact of outliers, and improved convergence during model fitting.

For descriptive statistics, continuous variables are summarized as mean ± standard deviation (M ± SD), while categorical variables are reported using absolute counts (*n*) and relative frequencies (%).

#### 2.6.1 RRi-vs-time model

All RRi data were preprocessed using a zero-phase Butterworth low-pass filter, which suppressed high-frequency fluctuations while preserving the slower underlying trends of interest. Ectopic heartbeats were then identified and removed by fitting a local regression to the RRi signal, computing the residuals, and excluding values whose residuals exceeded twice the median absolute deviation. This procedure was implemented with the CardioCurveR (v1.0.0) R package, specifically developed for this purpose (Castillo-Aguilar, 2025).

In order to obtain each individual’s exercise-induced CAS, we model the R–R intervals (RRi) across a complete rest-exercise-recovery protocol, as early described (Castillo-Aguilar et al., 2025). Under this specification, each observed RRi is assumed to follow a normal distribution centered on a time-dependent function. This data generation process is depicted in Equation 1.


$$\mathrm{RRi}∼N(f(t|\theta_{k}),\sigma^{2})$$ (1)

Here, $\sigma^{2}$ represents the variance term, and the function $f(t|\theta_{k})$ takes the form described in Equation 2.


$$f(t|\theta_{k})=\alpha +\underset{\text{Drop Phase}}{\underset{︸}{\frac{\beta }{1+e^{\lambda (t-\tau )}}}}-\underset{\text{Recovery Phase}}{\underset{︸}{\frac{\mathrm{c\beta}}{1+e^{\phi (t-\tau -\delta )}}}}$$ (2)

The parameter vector $\theta_{k}=\{\alpha ,\beta ,c,\lambda ,\phi ,\tau ,\delta \}$ governs the shape of the RRi trajectory over time. Specifically, the parameters α , β , and c are magnitude parameters: β controls the initial drop in RRi during exercise, cβ controls the subsequent recovery magnitude, and α serves as the baseline RRi level. The parameters λ and ϕ are rate parameters that determine the steepness (i.e., the speed) of the drop and recovery transitions, respectively. Finally, the timing parameters τ and δ indicate when the drop phase begins and how the recovery phase onset is shifted relative to τ .

#### 2.6.2 Two-step modeling strategy

Our modeling proceeded in two distinct stages. First, for each individual, we obtained cardiac-autonomic signature (RRi-vs-time, α,β,c,λ,ϕ,τ,δ ) using the R package *CardioCurveR* (v1.0.0). This package implements a box-constrained quasi-Newton algorithm with a Huber loss function for robust parameter estimation (Castillo-Aguilar, 2025).

Second, we specified two multivariate Bayesian regression models in which each invidual’s cardiac-autonomic signature component ( ${\hat{\theta }}_{\mathrm{ik}}$ ) served as a response. Concretely, for the k th parameter of subject i we let the conditional mean


$$\mu_{k,i}=\beta_{k,0}+\sum_{p=1}^{6}\beta_{k,p}x_{p,i}$$ (3)

where $x_{i1},\ldots ,x_{i6}$ denote the six centered and scaled immune-cell predictors (absolute lymphocyte count, total B-cell count, and the four B-cell subpopulations defined by CD21/CD11c expression). In the first model, we regressed ${\hat{\theta }}_{\mathrm{ik}}$ on those six immune predictors via Equation 3. In the second model, we extended Equation 3 by adding four additional terms, $\beta_{k,\mathrm{age}}({\mathrm{age}}_{i})$ , $\beta_{k,\mathrm{sex}}({\mathrm{sex}}_{i})$ , $\beta_{k,\mathrm{fat}}({\mathrm{fat}}_{i})$ and $\beta_{k,\text{muscle}}({\text{muscle}}_{i})$ , to account for confounding by age, sex and body composition variables when modeling cardiac-autonomic signatures. Prior to fitting either multivariate model, all continuous predictors (immune counts and age) were centered to their mean and scaled by their standard deviation. We specified N(0,3) priors on every main-effect coefficient $\beta_{k}$ to exert a weakly regularizing influence and to mitigate the impact of outliers (Lemoine, 2019).

#### 2.6.3 Model fitting and inference

Bayesian estimation employed the No-U-Turn Sampler (a variant of Hamiltonian Monte Carlo) as implemented in the *brms* (v2.22.0) and *rstan* (v2.32.7) packages in R (R Core Team, 2021). For each multivariate model, five Markov chains were run, each with 2,000 warm-up iterations followed by another 2,000 sampling iterations, resulting in 10,000 post-warmup samples per parameter.

Inference followed the SEXIT (Sequential Effect eXistence and Significance Testing) framework (Makowski et al., 2019). For each estimated parameter and its corresponding posterior distribution, we report the posterior median and its 95% highest-density credible interval (HDI). We also present the probability of direction (pd) as a quantitative measure of effect existence. Practical significance (ps) is indexed by the proportion of the posterior mass falling outside a region of practical equivalence (ROPE). The ROPE was defined as ±0.1 times the standard deviation of the response variable, and to ensure consistency all predictors were standardized prior to modeling (Makowski et al., 2019).

#### 2.6.4 Model diagnostics

To verify sampling convergence and stability, we checked that the potential scale reduction factor ( $\hat{R}$ ) for every parameter was below 1.01 and that the effective sample size exceeded 1,000. We also inspected trace plots visually to confirm adequate chain mixing and performed posterior predictive checks to ensure the model’s predicted RRi distributions aligned with the observed data. All analyses were fully implemented in R (R Core Team, 2021).

Further model diagnostics, parameters and posterior statistics are available in the Supplementary material.

## 3 Results

### 3.1 Sample characteristics

A total of 81 older adults were included (mean age 70.6 ± 5.8 years, range 61–89 years old), with 25 (30.9%) males and 56 (69.1%) females. Baseline demographic, anthropometric and immunological characteristics are summarized in Table 1.

**Table 1:** Baseline sociodemographical, body composition and immunological characteristics by sex.

		Sex			
Characteristic	Overall *N* = 81	Females *N* = 56	Males *N* = 25	Difference	95% CI
Age (years old)	70.60 ± 5.80	70.07 ± 5.66	71.80 ± 6.06	−0.30	−0.77, 0.17
Weight (kg)	74.69 ± 14.48	72.75 ± 14.68	79.16 ± 13.25	−0.47	−0.95, 0.02
Height (cm)	156.72 ± 8.46	153.11 ± 6.13	164.80 ± 7.34	−1.8	−2.3, −1.2
BMI (kg/m^2^)	30.48 ± 6.16	31.11 ± 6.69	29.03 ± 4.55	0.37	−0.11, 0.85
Muscle mass (kg)	45.19 ± 8.53	41.02 ± 4.86	54.52 ± 7.57	−2.2	−2.7, −1.6
Body fat (%)	35.35 ± 8.77	39.38 ± 6.46	26.32 ± 6.15	2.1	1.5, 2.7
Total lymphocytes (*n*/μL)	70,686.04 ± 27,256.28	74,011.47 ± 28,149.43	63,342.38 ± 24,115.72	0.41	−0.07, 0.90
Total B cells (*n*/μL)	9,752.43 ± 5,513.14	10,513.08 ± 5,581.69	8,072.67 ± 5,071.42	0.46	−0.02, 0.95
CD21^−^CD11c^+^ B cells (*n*/μL)	328 ± 218	340 ± 229	302 ± 191	0.18	−0.30, 0.67
CD21^−^CD11c^+^ B cells (%)	3.68 ± 2.26	3.41 ± 1.78	4.28 ± 3.03	−0.36	−0.84, 0.13
CD21^+^CD11c^+^ B cells (*n*/μL)	198 ± 139	202 ± 138	188 ± 142	0.10	−0.38, 0.58
CD21^+^CD11c^+^ B cells (%)	2.15 ± 1.33	1.96 ± 0.94	2.56 ± 1.89	−0.41	−0.90, 0.08
CD21^+^CD11c^−^ B cells (*n*/μL)	8,351 ± 4,994	8,961 ± 5,083	7,029 ± 4,624	0.40	−0.08, 0.89
CD21^+^CD11c^−^ B cells (%)	84.60 ± 11.57	84.05 ± 13.38	85.80 ± 6.11	−0.17	−0.66, 0.31
CD21^−^CD11c^−^ B cells (*n*/μL)	727 ± 488	807 ± 535	553 ± 306	0.59	0.10, 1.1
CD21^−^ CD11c^−^ B cells (%)	7.81 ± 3.84	8.02 ± 4.32	7.36 ± 2.53	0.19	−0.29, 0.67

Data is presented as mean ± standard deviation (SD). Differences are expressed as standardized mean differences (female–male) with 95% confidence intervals (95% IC).

The observed frequency of ABCs in our cohort is comparable to that reported by McGrath et al. (2024), suggesting potential context-dependent variation linked to population or disease status (McGrath et al., 2024). Our immunophenotypic classification of B-cell subsets aligns with previous studies, in which CD21^+^CD11c^−^ cells are typically naïve or central memory phenotypes, while CD21^−^CD11c^+^ and CD21^−^CD11c^−^ subsets are linked to activation, antigen experience, or anergic/exhausted states (Tangye and Tarlinton, 2009; Sosa-Hernández et al., 2022). These findings are consistent with earlier reports of ABC expansion in aging and autoimmune conditions, reinforcing their potential relevance as immunological biomarkers (Pinto et al., 2023).

A detailed characterization aggregated by age and sex can be observed in Supplementary Figure S1, found in section “B Cell Subpopulation Distribution” in the Supplementary material.

### 3.2 Exercise-induced cardiac autonomic trajectory

**Figure 2:**
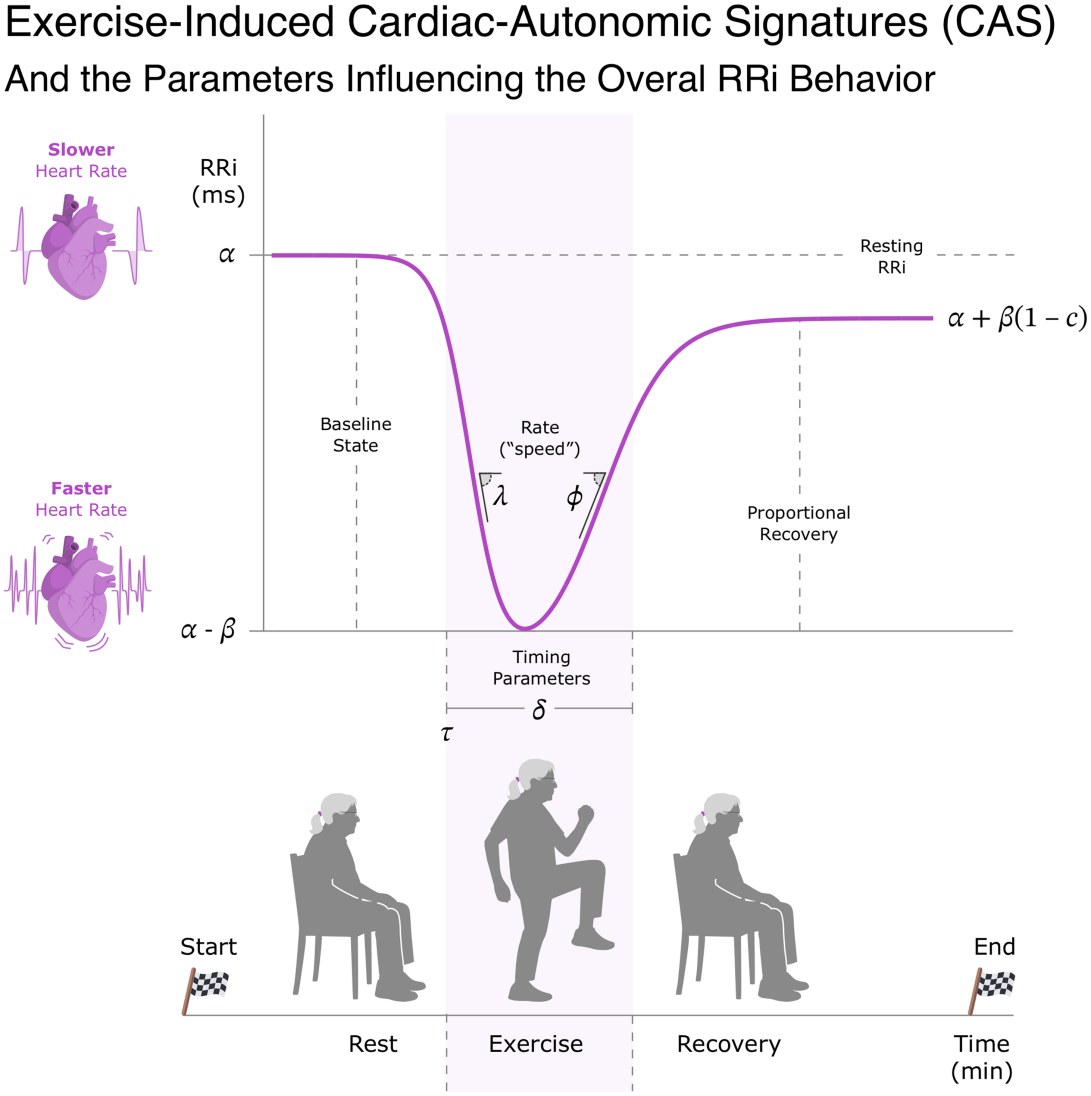
Cardiac autonomic signature given by RRI dynamics in response to exercise as described by the RRI-vs-time model. The image also depicts the rest-exercise-recovery protocol used to induce the autonomic changes in response to physical exertion. Parameters α,β,c control the RRI transient states, *φ*, λ,ϕ control the steepneses or speed of change in RRI transient states, *δ* and τ,δ control thde timing of these changes in RRI dynamics.

Exercise induced a characteristic U-shaped form on RRi during a rest-exercise-recovery protocol. This trajectory was parameterized using a coupled logistic RRi-vs-time model, with shape controlled by the model parameters shown in Figure 2.

In our sample, the baseline RRi controlled by α was 884.77 ms (CI_95%_[840.27, 925.77]), with an exercise induced drop ( β ) of 403.99 (CI_95%_[355.6, 451.58]). The recovery proportion ( c parameter) relative to β was 0.89 (CI_95%_[0.84, 0.94]).

In relation to the rate parameters, controlling the speed of transition between exercise and recovery phases, we found a rate of exercise-induced decay ( λ ) of 3.01 min^−1^ (CI_95%_[2.57, 3.44]) and a post exercise recovery rate ( ϕ ) of 2.18 min^−1^ (CI_95%_[1.63, 2.71]).

For time parameters controlling the timing of the aforementioned RRi kinetics, we observed that the time of the exercise-induced ( τ ) drop aligned at 6.89 (CI_95%_[6.66, 7.11]) and a duration of the exertion-related RRi depression ( δ ) was prolonged for 2.50 (CI_95%_[2.28, 2.72]).

### 3.3 B cell expression on cardiac autonomic-immune axis

**Figure 3:**
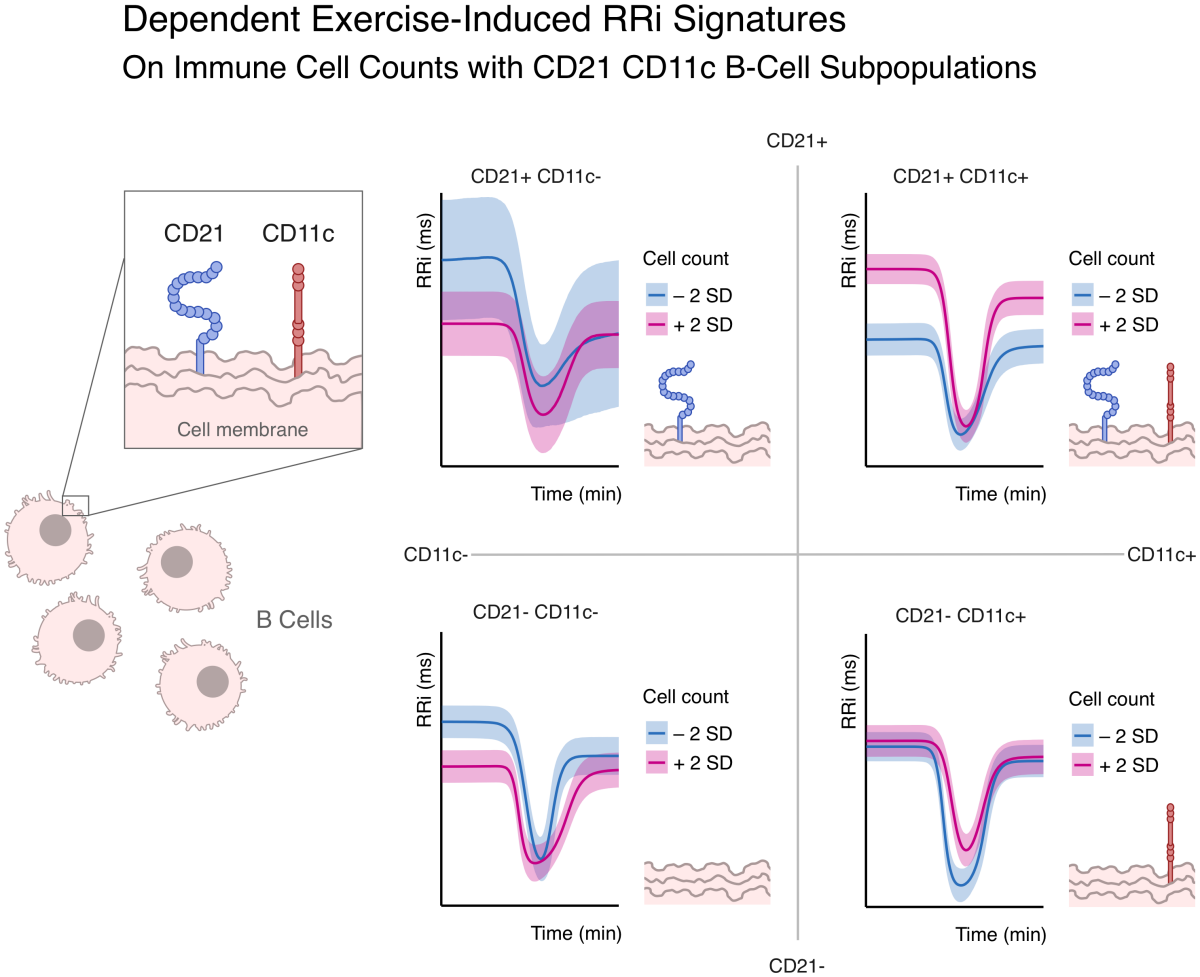
Predicted exercise-induced RRI signatures, dependent on CD21 and CD11c B cell expressions. Each RRI-vs-time curve correspond to the estimated Cardiac Autonomic Signature (line: median, shaded area: 95% high density interval, HDI) for the corresponding combination of B cell phenotypes (of CD21 and CD11c) below and above two standard deviations (blue and violet shaded areas), while keeping the remaining cell markers counts constant at their mean. Depicted effects are adjusted for confounding variables.

Exercise-induced Cardiac Autonomic Signature (CAS) showed distinct associations with the CD21/CD11c defined B cell subsets. The specific morphological changes of the RRi-vs-time curve were influenced by relative abundance of each B subpopulation, indicating that B cell phenotypes are linked to autonomic adaptability. The main immune-autonomic associations derived by different B cell subsets can be seen in Figure 3.

#### 3.3.1 CD21^+^/CD11c^−^

A higher proportion of CD21^+^CD11c^−^ B cells, typically representing naïve or central memory phenotypes, were associated with more than 80% posterior probability of having an attenuated RRi decrease (ES on β = −0.45, CI_95%_[−1.29, 0.32], pd. = 86%, ps = 80.2%), these individuals, also exhibit faster autonomic transition, as indicated by a steeper exercise-induced changes, both at onset (ES on λ = 0.43, CI_95%_[−0.41, 1.29], pd. = 83.7%, ps = 77.3%) and recovery phase (ES on ϕ = 0.49, CI_95%_[−0.36, 1.34], pd. = 87.8%, ps = 82.2%). Together, these associations suggest a profile of greater flexibility and preserved vagal adaptability in participants with higher proportions of this subset.

#### 3.3.2 CD21^+^/CD11c^+^

In contrast, CD21^+^CD11c^+^ B cells, linked to activated memory or antigen-experienced states, were strongly associated with a higher vagal resting tone (ES on α = 0.48, CI_95%_[0.11, 0.84], pd. = 99.5%, ps = 97.9%). However, this apparent advantage was coupled with greater RRi decline during exercise (ES on β = 0.34, CI_95%_[0, 0.71], pd. = 97.3%, ps = 91.5%), incomplete post-exercise RRi recovery (ES on c = −0.24, CI_95%_[−0.6, 0.1], pd. = 91.5%, ps = 79.1%) and prolonged RRi depression following exertion (ES on δ = 0.26, CI_95%_[−0.12, 0.67], pd. = 90.5%, ps = 79.5%). This suggests that despite higher resting tone, these participants may recruit autonomic responses less efficiently under stress, with worse recovery capacity. This pattern reflects a state of initially higher vagal activity, but reduced resilience, suggesting impaired ability to restore autonomic balance after stress.

#### 3.3.3 CD21^−^/CD11c^−^

The CD21^−^CD11c^−^ subset is often associated with anergic or exhausted B cells. Higher proportions of this subset were associated with lower baseline vagal tone (ES on α = −0.31, CI_95%_[−0.7, 0.09], pd. = 93.5%, ps = 84.7%), but also with a less marked exercise-induced RRi drop (ES on β = −0.41, CI_95%_[−0.8, −0.02], pd. = 98.2%, ps = 94.2%), and more complete post-exercise recovery (ES on c = 0.34, CI_95%_[−0.04, 0.72], pd. = 96.1%, ps = 89.2%). In addition, they demonstrated a steeper RRi drop (ES on λ = 0.44, CI_95%_[−0.01, 0.84], pd. = 98%, ps = 94.4%) and early autonomic engagement (ES on τ = −0.3, CI_95%_[−0.68, 0.14], pd. = 92.8%, ps = 83.5%).

#### 3.3.4 CD21^−^/CD11c^+^

Finally, CD21^−^CD11c^+^ (ABCs), were associated with a less steep RRi drop during exertion (ES on λ = −0.24, CI_95%_[−0.61, 0.13], pd. = 90.2%, ps = 76.8%) and delayed onset of autonomic engagement (ES on τ = 0.32, CI_95%_[−0.04, 0.68], pd. = 95.9%, ps = 88.7%). representing a phenotype characterized for slower and delayed autonomic adjustments to exercise.

**Figure 4:**
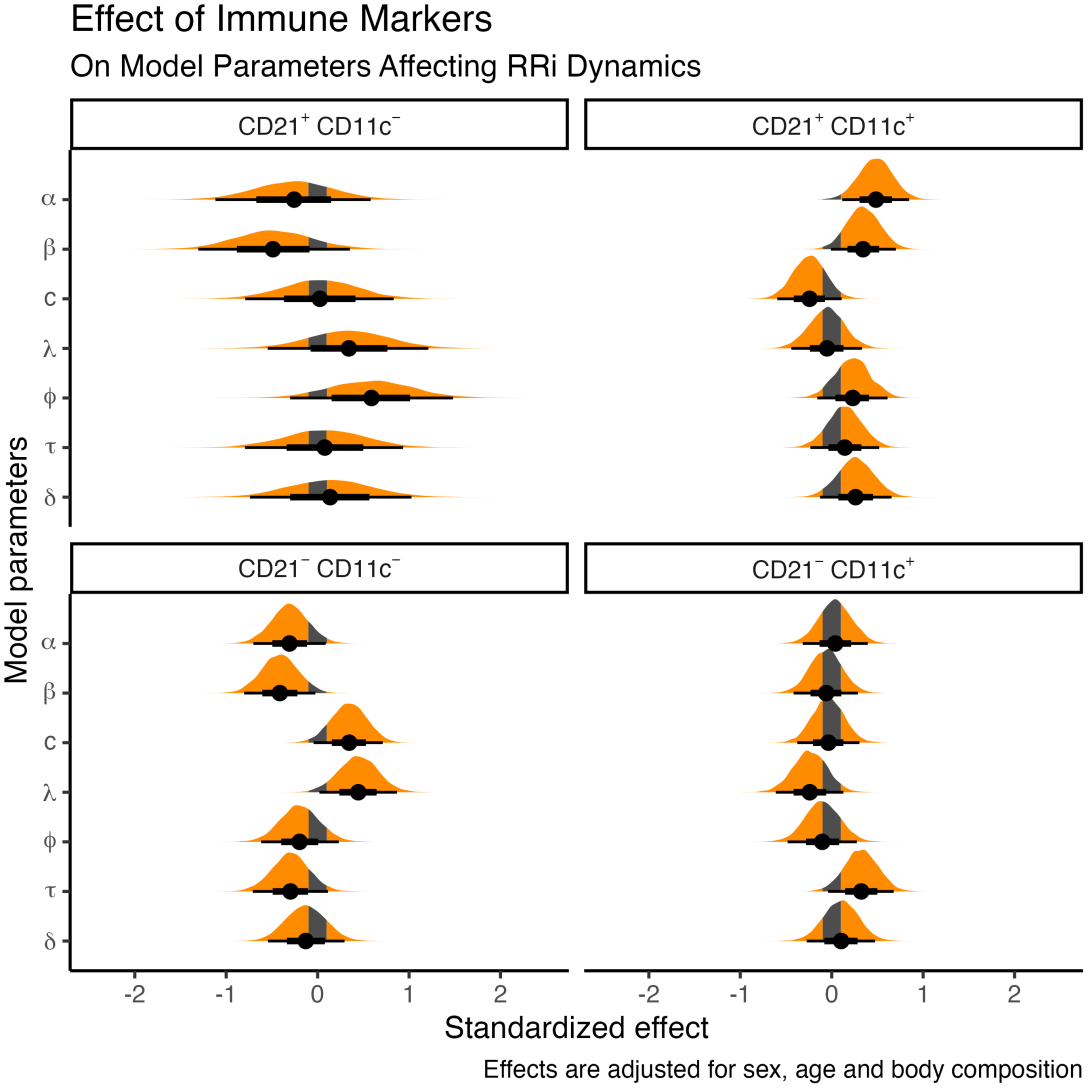
Posterior distribution of the standardized effect of B cell CD21 and φCD11c expression on model parameters influencing the exercise-induced cardiac autonomic dynamics. α controls the baseline RRi; β is proportional to the RRi drop, induced by exercise; c is the recovery proportion, relative to β ; λ and ϕ are the steepness of exercise-induced RRi drop and post exercise recovery; τ correspond to the time at which the exercise-induced RRi drop occurs; δ correspond to the time-duration of the RRi depression, relative to τ , that takes before the recovery kinetics begin. Shaded areas correspond to the region of practical equivalence (ROPE).

These results provide compelling evidence that distinct B cell phenotypes, defined by CD21/CD11c expression, differentially modulate exercise-induced autonomic responses. Naïve/central memory cells were linked to more flexible and adaptative profiles, activated memory cells to incomplete recovery, anergic/exhausted cells to efficient stress handling despite lower resting vagal tone, and ABCs to delayed responses to effort. Figure 4 illustrates posterior distributions of standardized effects for each parameter, highlighting the nuanced interplay between immune phenotype and autonomic function controlling the exercise-induced RRi dynamics.

## 4 Discussion

In this cohort of older adults, B-cell subpopulations defined by CD21/CD11c expression showed distinct associations with exercise-induced cardiac autonomic signatures (CAS). Individuals with a higher proportion of CD21^+^/CD11c^+^ B cells exhibited characteristics of heightened parasympathetic activity at rest, including elevated baseline vagal tone with longer resting RR intervals (greater α ). This group also displayed an increased sympathetic response during exercise, indicated by an abrupt drop in Rri (greater β ), a decreased cardiac recovery post-exercise (lower c ) and a longer time-to-recovery began (greater δ ). Conversely, participants with a predominance of CD21^−^/CD11c^−^ B cells showed the opposite autonomic phenotype: low baseline vagal tone characterized by shorter RR and intervals (lower α ). They also presented a decreased sympathetic response to physical exertion, reflected in a smaller (lower β ), but steeper, drop in Rri (higher λ ), and a more close-to-baseline heart rate return following exercise (higher c ). These contrasting observations suggest that the relative abundance of these B cell subpopulations may serve as an indicator of an individual’s autonomic balance, with the CD21^+^/CD11c^+^ phenotype aligning with a vagally dominant state with increased sympathetic reactivity, while the CD21^−^/CD11c^−^ phenotype corresponds to a more sympathetically inclined state coupled with efficient compensatory vagal recovery capacity.

### 4.1 CD21^+^/CD11c^+^ subpopulation: increased vagal tone and modulated sympathetic reactivity

The association of a higher proportion of CD21^+^/CD11c^+^ B cells, linked to activated memory or antigen-experienced states, with elevated resting parasympathetic tone is consistent with the established benefits of higher vagal activity, including lower systemic inflammation and improved health status (Olivieri et al., 2024). This vagotonic profile, often marked by increased HRV, has been shown to correlate inversely with pro-inflammatory cytokines (Marsland et al., 2007; Arias-Colinas et al., 2022), suggesting a more regulatory immunological microenvironment potentially influenced by the cholinergic anti-inflammatory reflex (Tracey, 2002; Giunta et al., 2024). Furthermore, there’s evidence suggesting that vagal signaling appears to promote IgA production and maintain barrier immune homeostasis (McLoughlin et al., 1976), possibly through the preferential localization of CD21^+^/CD11c^+^ memory B cells in lymphoid tissues with rich cholinergic innervation, such as the spleen and lymph nodes (Bonaz et al., 2017), where acetylcholine (ACh) could modulate their functions towards a more anti-inflammatory and regulatory phenotype (Jacobson et al., 2021).

The increased sympathetic response during exercise observed in this group is another notable finding. Moreover, a high resting vagal tone typically leads to a more pronounced vagal withdrawal at the onset of exercise, which aligns well with the observed data, suggesting a more pronounced initial heart rate acceleration, potentially due to increased vagal withdrawal and cholinergic modulation of the autonomic nervous system (ANS) (Giunta et al., 2024). Cholinergic activation has been shown to counteract sympathetic signals, which might limit the exercise-induced mobilization of lymphocytes (Smith et al., 2025), potentially preventing marked immune activation in response to physical stress, thus suggesting a more controlled immune state in individuals with a dominant vagal influence.

However, this vagal-centric profile was also characterized by a slower return to baseline heart rate after exercise. Despite a less pronounced initial increase in heart rate, individuals with more CD21^+^/CD11c^+^ B cells took longer to recover at the same extent that individuals with greater CD21^−^/CD11c^−^. This protracted recovery could stem from a less efficient re-establishment of autonomic balance following a moderated sympathetic response, where the sympathetic nervous system (SNS) might not have been sufficiently activated to trigger a strong subsequent vagal rebound. Alternatively, it might indicate a degree of autonomic rigidity (Castillo-Aguilar et al., 2025), where a predominantly parasympathetic system at rest exhibits difficulty in rapidly adjusting after a perturbation. The lower c parameter (recovery proportion of the RRi curve) observed in this group supports the notion of reduced autonomic flexibility, suggesting that while a basal anti-inflammatory environment might be present, the capacity for rapid adaptation to acute physiological challenges could be limited.

The characteristics associated with a higher proportion of CD21^+^/CD11c^+^ B cells find parallels in active older adults, who often display high resting HRV and moderated cardiovascular responses to exercise (Mabe-Castro et al., 2024), along with less inflammatory immune profiles (Tylutka et al., 2021). This suggests that these memory B cells might serve as both a marker and a modulator of this physiological state. While CD11c^+^ B cells in healthy individuals can differentiate into plasmablasts upon stimulation (Golinski et al., 2020), they also exhibit some resistance to regulatory signals like IL-10 (Golinski et al., 2020), implying potential modulation by neuroendocrine pathways. Future research into the effects of interventions such as chronic vagal nerve stimulation on these cells could be valuable in exploring ways to enhance their anti-inflammatory profile while preserving their capacity for immune response when needed.

### 4.2 CD21^−^/CD11c^−^ subpopulation: sympathetic predominance and compensatory response

In contrast, older adults with a higher proportion of CD21^−^/CD11c^−^ B cells, often associated with anergic or exhausted B cells, presented an autonomic profile indicative of sympathetic predominance at rest and during exercise, coupled with an efficient vagal recovery post-exercise. The low baseline vagal tone observed in this group supports the idea that these double negative B cells are associated with a microenvironment of chronic immune stimulation and inflammation, as reduced CD21 expression is often a hallmark of anergic or exhausted B cells resulting from continuous antigenic stimulation (Del Padre et al., 2020), conditions frequently linked with chronic sympathetic activation (Giunta et al., 2024). Our findings align with studies demonstrating that chronically low HRV in older individuals is associated with higher levels of pro-inflammatory cytokines (Jiang et al., 2022), suggesting that an accumulation of CD21^−^ B cells tends to coexist with sympathetic dominance and inflammaging. Prolonged exposure to pro-inflammatory signals can induce a senescence-like phenotype in B cells (Frasca et al., 2023), further suggesting that a predominance of CD21^−^/CD11c^−^ B cells may reflect chronic immunometabolic stress and related sympathetic overactivation.

During exercise, this subpopulation exhibited a faster autonomic response, with the marked tachycardia, reflected in a greater λ and lower α parameters, indicating a robust sympathetic discharge. This heightened response is consistent with their low baseline parasympathetic tone; with less vagal “braking,” the cardiovascular system responds rapidly to the exercise stimulus. While potent acute sympathetic activation has advantages such as increased cardiac output and immune cell mobilization (Smith et al., 2025), potentially contributing to the clearance of senescent cells through exercise (Simpson et al., 2021), it also carries potential risks, including cardiac arrhythmias and increased inflammation.

A notable and potentially beneficial finding was the better autonomic recovery (high c parameter) observed in the CD21^−^/CD11c^−^ group despite their lower initial vagal tone. This suggests a compensatory and efficient vagal reactivation following the sympathetic surge, possibly mediated by baroreceptor and mechanoreceptor reflexes responding to the exercise-induced cardiovascular changes (Besnier et al., 2017). This efficient cholinergic recovery could aid in resolving immune activation once the exercise stimulus ceases. Thus, individuals with a more sympathicotonic profile might be capable of mounting strong but transient responses, followed by a relatively rapid return to autonomic balance mediated by the parasympathetic nervous system. This pattern resonates with descriptions of mild autonomic insufficiency, where sympathetic hyper-responsiveness can coexist with late vagal hyper-reactivity (Besnier et al., 2017), and aligns with the concept of autonomic resilience (Castillo-Aguilar et al., 2025).

This autonomic and immune profile shares similarities with clinical conditions such as metabolic syndrome and obesity, where reduced HRV and high basal sympathetic activity are often observed, although some form of interventions can improve vagal reactivity (Tylutka et al., 2021; Frasca et al., 2023). The link between alterations in B cells and autonomic behavior suggests a bidirectional relationship, where not only can the immune profile influence the autonomic response, but also that modulating the ANS can potentially impact age-associated immune alterations, as seen with chronic exercise interventions (Weyh et al., 2020; Tylutka et al., 2021).

### 4.3 Strengths and limitations

This study integrates autonomic physiology and detailed B-cell phenotyping in healthy aging by applying nonlinear modeling of RRi dynamics within a coupled logistic framework to capture temporally resolved autonomic shifts during exercise and recovery, providing a more dynamic assessment than standard HRV metrics, while controlling for key demographic and body-composition covariates. Using surface-marker definitions of CD21/CD11c B-cell subsets, we observed distinct associations between those subpopulations and specific autonomic parameters, suggesting novel immune–ANS interactions and motivating mechanistic follow-up.

Several limitations temper interpretation. The cross-sectional design precludes causal inference; surface marker phenotyping identifies subpopulations but not their functional or mechanistic roles; and we lacked complementary functional assays or transcriptomic profiling, with potential unquantified batch/gating effects. Methodologically, our two-stage modeling pipeline did not propagate posterior uncertainty between steps, indicating the need for fully joint hierarchical inference. Because CD21/CD11c subsets are mutually exclusive, changes in one imply reciprocal changes in others, yet the Figure 3 simulations show theoretical contrasts with a single subpopulation varied while others held at their mean, which aids interpretation but is not biologically realistic; future work should therefore adopt compositional or constraint-based models, longitudinal designs, and functional/mechanistic assays to resolve causality and the biological roles of the identified B-cell populations in immune–autonomic aging.

## 5 Conclusion

This research suggests that B cell subpopulations reflect and potentially modulate ANS dynamics during exercise in older adults. This integrative neuro-immunological approach provides a more comprehensive understanding of the health status of older individuals, identifying “pro-resilient” versus “pro-fragile” profiles that might not be evident when evaluating systems separately. Individuals with a predominance of CD21^+^/CD11c^+^ B cells exhibit higher baseline vagal tone, an stronger sympathetic response during exercise, but an incomplete autonomic recovery. In contrast, those with CD21^−^/CD11c^−^ B cells present lower baseline vagal tone, a faster sympathetic driven response to exertion, and more complete post-exercise recovery. These results indicate that CD21^+^/CD11c^+^ B cells are associated with a more regulated and less inflammatory autonomic profile, whereas CD21^−^/CD11c^−^ B cells reflect a state of chronic inflammation. Exercise could modulate these autonomic and immune profiles, thereby improving cardiovascular health in older adults. These findings highlight B cell subsets as potential biomarkers of autonomic adaptability in aging, and suggest that exercise interventions could modulate these neuro-immunological interactions to promote cardiovascular health.

## Data Availability

The original contributions presented in the study are included in the article/Supplementary material, further inquiries can be directed to the corresponding author/s.
