# Supplementary material for: Immune-derived cardiac autonomic signatures: predicting autonomic responses to exercise from B-cell phenotypes
Source: Front Neurosci. 2026 Jan 15;19:1702281. doi: 10.3389/fnins.2025.1702281 (PMC12851968; doi:10.3389/fnins.2025.1702281)
Supplement: Supplementary file 1 [file Data_Sheet_1.DOCX]

Supplementary Material

**Matías Castillo-Aguilar^1,2^, Ginés Viscor^3^, Lindybeth Sarmiento^1,2^, Julieta Sepúlveda^1,4^, Marcelo Navarrete^1,2^, Cristian Núñez-Espinosa^1,2^ ***

^1^ Centro Asistencial Docente y de Investigación (CADI-UMAG), Punta Arenas, Chile.

^2^ Escuela de Medicina, Universidad de Magallanes (UMAG), Punta Arenas, Chile.

^3^ Physiology Section, Department of Cell Biology, Physiology and Immunology, Faculty of Biology, Universitat de Barcelona, Barcelona, Spain.

^4^ Facultad de Ciencias de la Salud, Universidad de Magallanes (UMAG), Punta Arenas, Chile.

***Correspondence:**

Cristian Núñez-Espinosa, Escuela de Medicina, Universidad de Magallanes, Punta Arenas, Chile. Centro Asistencial de Docencia e Investigación CADI-UMAG, Chile. e-mail: [cristian.nunez@umag.cl](mailto:cristian.nunez@umag.cl). Address: Avenida Bulnes 01855, Box 113-D. Phone: +56 61 2201411.

**Keywords: B Cells; Heart Rate Variability; Autonomic Nervous System; Aging; Exercise; Immunosenescence.**

# B Cell Subpopulation Distribution

To explore age-associated changes in B cell subsets, we analyzed the absolute number of four defined subpopulations across age quartiles. The predominant subset in all groups was CD21⁺CD11c⁻ cells, with the highest mean count observed in the youngest quartile (Q1: 9,855 ± 5,198) and a slight decline in older groups (Q4: 7,479 ± 5,686). The CD21⁻CD11c⁺ subset, often associated with age-related or atypical B cells, showed a modest decrease with age (Q1: 774 ± 427 vs. Q4: 581 ± 412). Similarly, CD21⁺CD11c⁺ cells also declined across quartiles (Q1: 362 ± 254 vs. Q4: 292 ± 223). The smallest population, CD21⁻CD11c⁻, exhibited a consistent but modest reduction in cell count with age (Q1: 261 ± 182 vs. Q4: 131 ± 61). These findings suggest a gradual reshaping of the peripheral B cell compartment with aging, characterized by a relative decline of CD21-expressing subsets. Aggregated absolute cell counts by age quartiles and sex can be Figure S1.


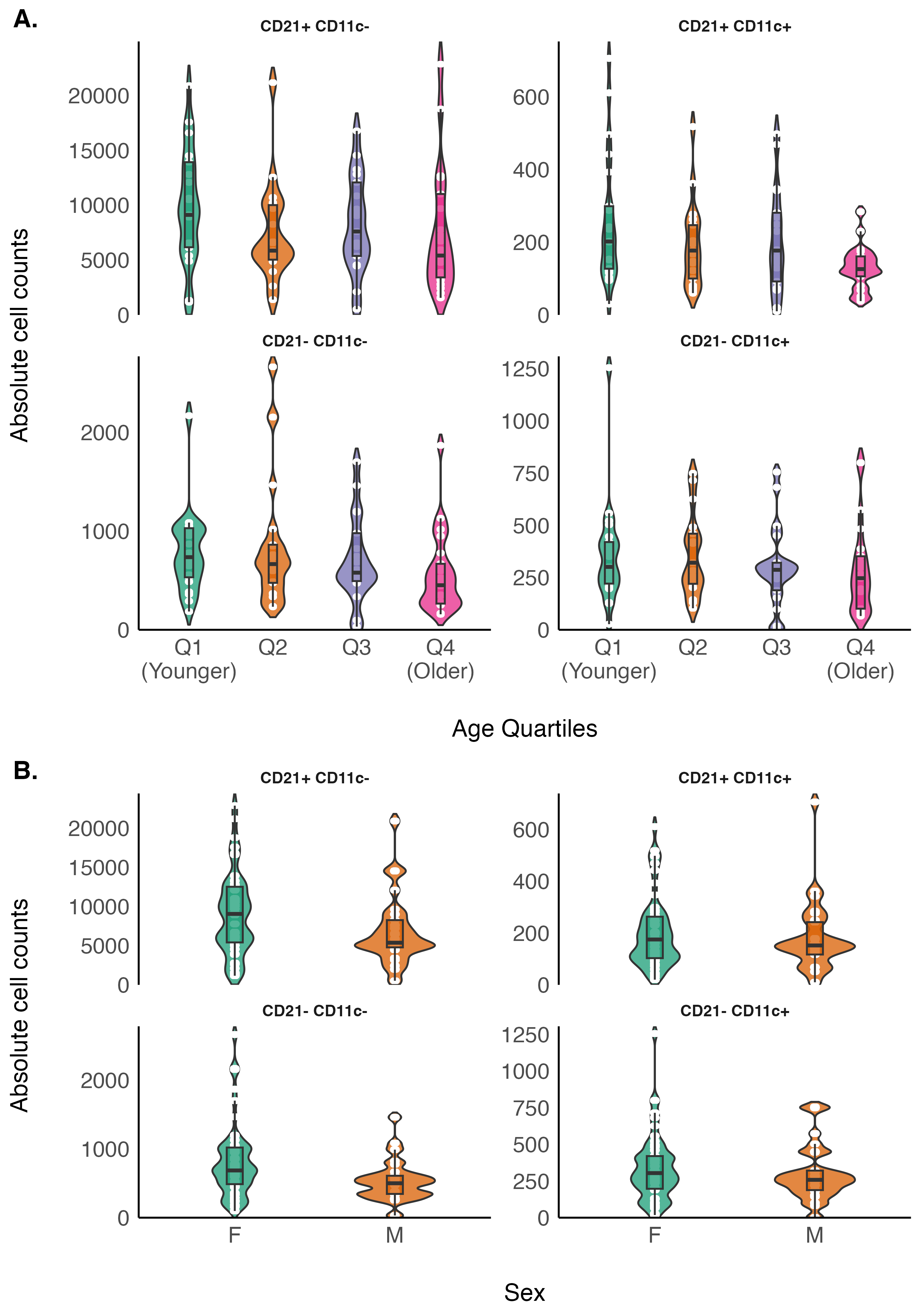


**Figure S1**. Distribution of B cell subpopulation counts across age quartiles (A) and sex (B). Each dot represents an individual; boxes indicate the interquartile range (IQR), and whiskers denote 1.5×IQR. Counts are shown for four phenotypic B cell subsets defined by CD21 and CD11c expression. F, female; M, male.

# Characterization of Model Estimates

In the following sections, we present the model estimates associated with the neuro-immune interactions and its effects on exercise-induced cardiac autonomic modulation.

## Unadjusted effects

First, we fitted a simple model without covariates. The standardized effects on the model parameters controlling the observed cardiac autonomic response to exercise can be seen in Figure S2 and Table S1, respectively.


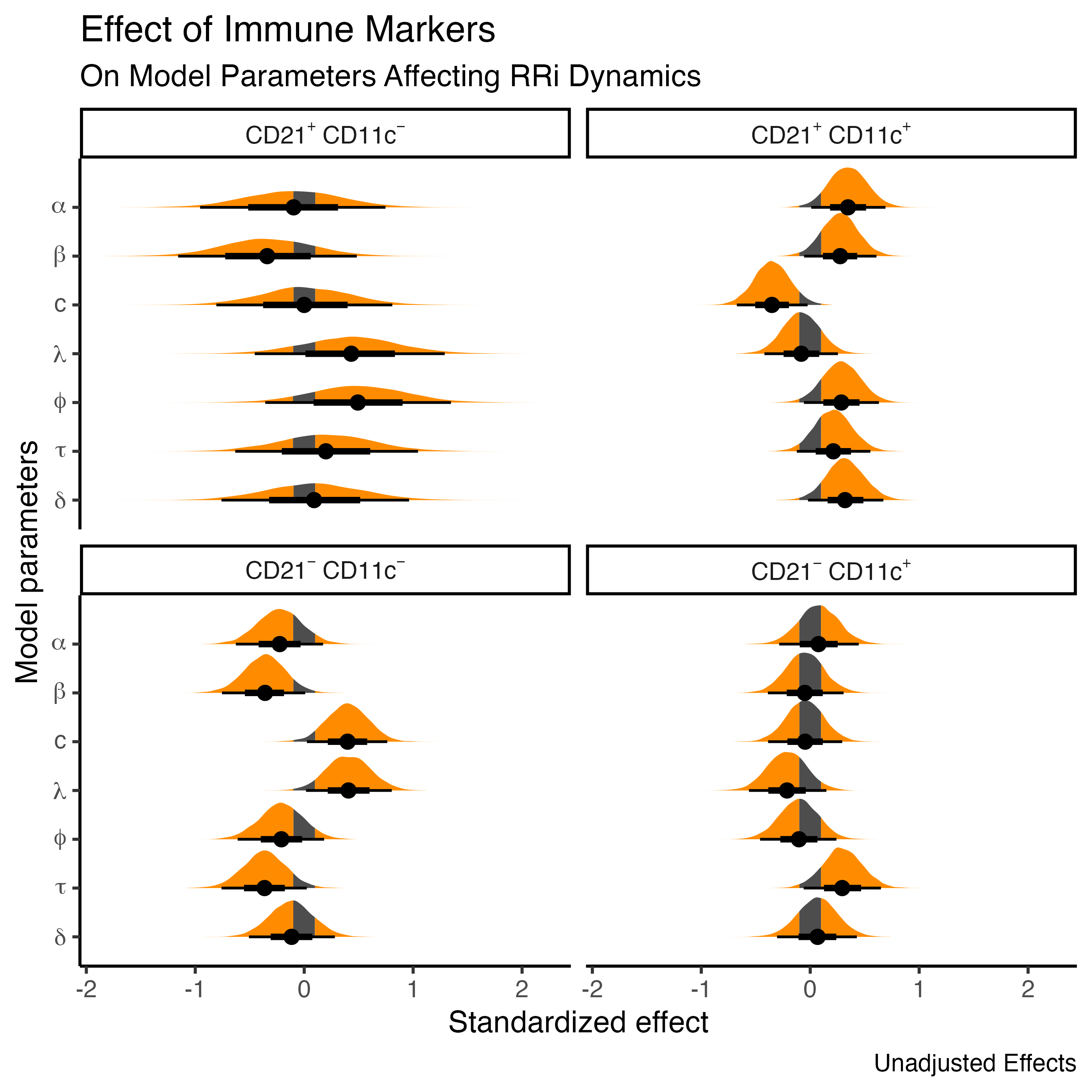
 **Figure S2**. Posterior distribution of the simple model on the parameter values associated with the exercise-induced cardiac autonomic response and the associated effects of B-cell phenotypes quantities, CD21 and CD11c. The shaded area correspond to the range of practical equivalence (ROPE), denoted as 0.1 standardized units. $\alpha$, denotes the baseline RRi level at rest; $\beta$, denotes the exercise-induced drop in the RRi; $c$, denotes the recovery proportion, relative to $\beta$; $\lambda$ and $\phi$ denote the exercise-induced drop and recovery rate paramters; $\tau$ and $\delta$ denote the timing parameters, that controls when the drop occurs and the time duration of the exercise-induced depression on RRi.

**Table S1**. Model estimates on the parameter values associated with the exercise-induced cardiac autonomic response and the associated effects of total, B and CD21 and CD11c B lymphocytes. Model effects are displayed unadjusted. *alpha*, denotes the baseline RRi level at rest; *beta*, denotes the exercise-induced drop in the RRi; *c*, denotes the recovery proportion, relative to *beta*; *lambda* and *phi* denote the exercise-induced drop and recovery rate paramters; *tau* and *delta* denote the timing parameters, that controls when the drop occurs and the time duration of the exercise-induced depression on RRi.

| Parameter | Estimate | 95% CI | PD | PS | ESS | R-hat |
| --- | --- | --- | --- | --- | --- | --- |
| alpha Total lymphocytes | -0.07 | [-0.42, 0.28] | 0.659 | 0.442 | 9163.8 | 1.000 |
| alpha Total B lymphocytes | 0.00 | [-0.98, 0.99] | 0.503 | 0.425 | 6196.1 | 1.000 |
| alpha CD21 - CD11C + | 0.08 | [-0.28, 0.45] | 0.663 | 0.450 | 9038.2 | 1.000 |
| alpha CD21 - CD11C - | -0.22 | [-0.61, 0.19] | 0.866 | 0.731 | 9782.8 | 1.000 |
| alpha CD21 + CD11C - | -0.10 | [-0.95, 0.75] | 0.586 | 0.498 | 6537.2 | 1.000 |
| alpha CD21 + CD11C + | 0.35 | [0.02, 0.7] | 0.978 | 0.926 | 11549.1 | 1.000 |
| beta Total lymphocytes | 0.15 | [-0.2, 0.49] | 0.797 | 0.613 | 7131.4 | 1.000 |
| beta Total B lymphocytes | 0.30 | [-0.65, 1.23] | 0.730 | 0.657 | 4605.8 | 1.001 |
| beta CD21 - CD11C + | -0.05 | [-0.39, 0.3] | 0.611 | 0.387 | 7563.1 | 1.000 |
| beta CD21 - CD11C - | -0.36 | [-0.74, 0.01] | 0.972 | 0.920 | 7557.4 | 1.000 |
| beta CD21 + CD11C - | -0.34 | [-1.14, 0.49] | 0.791 | 0.715 | 4802.4 | 1.001 |
| beta CD21 + CD11C + | 0.27 | [-0.04, 0.63] | 0.951 | 0.854 | 8205.1 | 1.000 |
| c Total lymphocytes | -0.25 | [-0.6, 0.07] | 0.930 | 0.810 | 10493.0 | 1.000 |
| c Total B lymphocytes | 0.21 | [-0.76, 1.09] | 0.668 | 0.591 | 7186.2 | 1.000 |
| c CD21 - CD11C + | -0.05 | [-0.39, 0.29] | 0.606 | 0.378 | 10547.0 | 1.000 |
| c CD21 - CD11C - | 0.40 | [0.04, 0.78] | 0.980 | 0.943 | 9634.2 | 1.000 |
| c CD21 + CD11C - | 0.00 | [-0.8, 0.82] | 0.500 | 0.405 | 7627.7 | 1.000 |
| c CD21 + CD11C + | -0.35 | [-0.68, -0.03] | 0.983 | 0.936 | 13335.0 | 1.000 |
| lambda Total lymphocytes | 0.17 | [-0.19, 0.52] | 0.820 | 0.642 | 7770.4 | 1.000 |
| lambda Total B lymphocytes | -0.63 | [-1.6, 0.38] | 0.889 | 0.848 | 4900.1 | 1.000 |
| lambda CD21 - CD11C + | -0.21 | [-0.56, 0.15] | 0.882 | 0.732 | 7625.5 | 1.000 |
| lambda CD21 - CD11C - | 0.41 | [0.02, 0.8] | 0.980 | 0.941 | 8047.0 | 1.000 |
| lambda CD21 + CD11C - | 0.43 | [-0.47, 1.27] | 0.836 | 0.778 | 4925.6 | 1.000 |
| lambda CD21 + CD11C + | -0.08 | [-0.41, 0.27] | 0.683 | 0.459 | 9818.9 | 1.000 |
| phi Total lymphocytes | 0.08 | [-0.3, 0.42] | 0.663 | 0.449 | 7597.5 | 1.000 |
| phi Total B lymphocytes | -0.48 | [-1.38, 0.58] | 0.833 | 0.776 | 4886.5 | 1.000 |
| phi CD21 - CD11C + | -0.10 | [-0.47, 0.23] | 0.723 | 0.508 | 8408.5 | 1.001 |
| phi CD21 - CD11C - | -0.21 | [-0.61, 0.18] | 0.853 | 0.712 | 7951.7 | 1.000 |
| phi CD21 + CD11C - | 0.49 | [-0.39, 1.31] | 0.876 | 0.822 | 5109.7 | 1.000 |
| phi CD21 + CD11C + | 0.29 | [-0.05, 0.63] | 0.949 | 0.857 | 10068.2 | 1.000 |
| tau Total lymphocytes | -0.16 | [-0.5, 0.2] | 0.817 | 0.640 | 10399.3 | 1.000 |
| tau Total B lymphocytes | -0.22 | [-1.18, 0.76] | 0.683 | 0.603 | 6994.5 | 1.000 |
| tau CD21 - CD11C + | 0.29 | [-0.06, 0.65] | 0.949 | 0.864 | 9999.5 | 1.000 |
| tau CD21 - CD11C - | -0.36 | [-0.75, 0.04] | 0.967 | 0.914 | 10774.0 | 1.000 |
| tau CD21 + CD11C - | 0.20 | [-0.65, 1.03] | 0.683 | 0.595 | 7451.7 | 1.000 |
| tau CD21 + CD11C + | 0.21 | [-0.12, 0.55] | 0.894 | 0.744 | 12680.7 | 1.000 |
| delta Total lymphocytes | -0.15 | [-0.52, 0.2] | 0.798 | 0.616 | 8307.9 | 1.000 |
| delta Total B lymphocytes | -0.14 | [-1.09, 0.91] | 0.612 | 0.535 | 5465.8 | 1.000 |
| delta CD21 - CD11C + | 0.07 | [-0.29, 0.44] | 0.646 | 0.429 | 8781.2 | 1.000 |
| delta CD21 - CD11C - | -0.12 | [-0.51, 0.28] | 0.724 | 0.533 | 8834.1 | 1.000 |
| delta CD21 + CD11C - | 0.09 | [-0.8, 0.92] | 0.587 | 0.491 | 5562.1 | 1.001 |
| delta CD21 + CD11C + | 0.32 | [-0.03, 0.66] | 0.966 | 0.903 | 11908.7 | 1.000 |

Moreover, the unadjusted residual correlations between model parameters are depicted in Table S2.

**Table S2**. Estimates of the residual correlation between model parameters controlling the exercise-induced cardiac autonomic response. Model effects are displayed unadjusted. *alpha*, denotes the baseline RRi level at rest; *beta*, denotes the exercise-induced drop in the RRi; *c*, denotes the recovery proportion, relative to *beta*; *lambda* and *phi* denote the exercise-induced drop and recovery rate paramters; *tau* and *delta* denote the timing parameters, that controls when the drop occurs and the time duration of the exercise-induced depression on RRi.

| Parameter | Estimate | 95% CI | PD | PS | ESS | R-hat |
| --- | --- | --- | --- | --- | --- | --- |
| alpha ~ beta | 0.32 | [0.11, 0.52] | 0.998 | 0.977 | 9406.5 | 1.000 |
| alpha ~ c | 0.01 | [-0.22, 0.24] | 0.542 | 0.229 | 12585.2 | 1.000 |
| beta ~ c | -0.17 | [-0.38, 0.07] | 0.926 | 0.734 | 11845.4 | 1.000 |
| alpha ~ lambda | 0.24 | [0.02, 0.45] | 0.981 | 0.884 | 9363.2 | 1.000 |
| beta ~ lambda | -0.34 | [-0.53, -0.14] | 0.998 | 0.984 | 11237.1 | 1.000 |
| c ~ lambda | 0.05 | [-0.18, 0.27] | 0.663 | 0.329 | 11930.4 | 1.000 |
| alpha ~ phi | 0.19 | [-0.03, 0.39] | 0.951 | 0.783 | 10077.6 | 1.000 |
| beta ~ phi | -0.46 | [-0.63, -0.27] | 1.000 | 1.000 | 10426.2 | 1.000 |
| c ~ phi | -0.21 | [-0.42, 0.02] | 0.965 | 0.842 | 9972.1 | 1.000 |
| lambda ~ phi | 0.23 | [0.01, 0.44] | 0.977 | 0.872 | 10711.1 | 1.000 |
| alpha ~ tau | -0.13 | [-0.35, 0.11] | 0.867 | 0.602 | 11817.4 | 1.000 |
| beta ~ tau | 0.06 | [-0.17, 0.28] | 0.684 | 0.363 | 11761.0 | 1.000 |
| c ~ tau | 0.05 | [-0.17, 0.29] | 0.652 | 0.327 | 11352.3 | 1.000 |
| lambda ~ tau | -0.31 | [-0.51, -0.1] | 0.997 | 0.970 | 12348.7 | 1.000 |
| phi ~ tau | -0.11 | [-0.33, 0.12] | 0.815 | 0.516 | 11315.5 | 1.000 |
| alpha ~ delta | 0.06 | [-0.17, 0.28] | 0.689 | 0.353 | 11341.4 | 1.000 |
| beta ~ delta | -0.26 | [-0.47, -0.04] | 0.988 | 0.921 | 11123.5 | 1.000 |
| c ~ delta | -0.12 | [-0.35, 0.11] | 0.838 | 0.550 | 11009.2 | 1.000 |
| lambda ~ delta | 0.39 | [0.18, 0.57] | 1.000 | 0.995 | 10059.0 | 1.000 |
| phi ~ delta | 0.04 | [-0.19, 0.26] | 0.632 | 0.303 | 11827.1 | 1.000 |
| tau ~ delta | -0.18 | [-0.4, 0.04] | 0.941 | 0.763 | 9128.6 | 1.001 |

The predicted cardiac autonomic kinetics to the full rest-exercise-recovery dynamics can be observed in Figure S2.


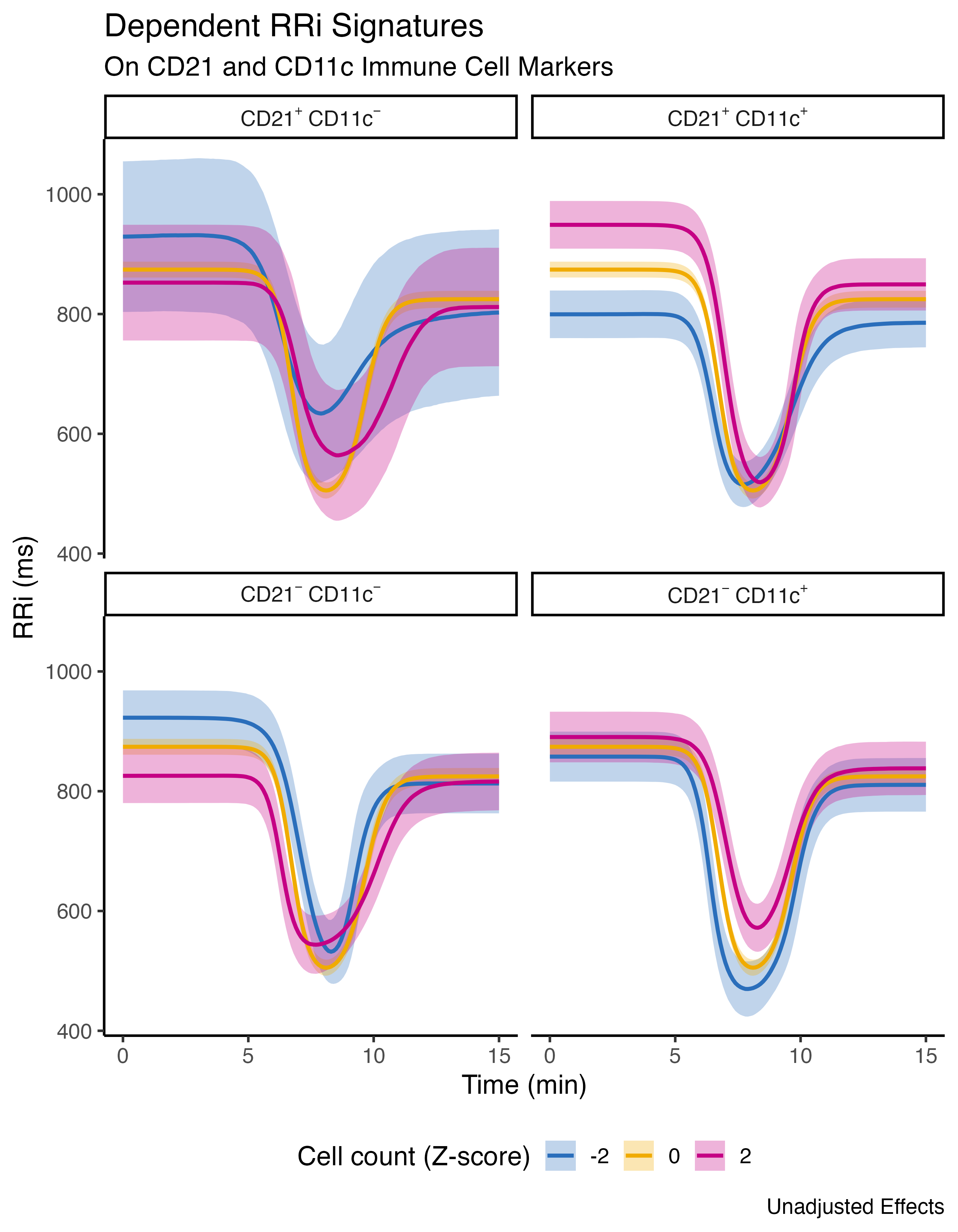
 **Figure S3**. Predicted cardiac autonomic signatures display different patterns dependent on the amounts of CD21 and CD11c B cell phenotypes (standardized units) and their corresponding cell subsets. These predicted dynamics are unadjusted.

## Adjusted effects

Finally, the last fitted model was adjusted considering the influence of sex age, and body composition as covariates. The adjusted standardized effects on the model parameters controlling the observed cardiac autonomic response to exercise can be seen in Table S3, respectively.

**Table S3**. Model estimates on the parameter values associated with the exercise-induced cardiac autonomic response and the associated effects of total, B and CD21 and CD11c B lymphocytes. Model effects are displayed adjusted by sex, age and body composition. *alpha*, denotes the baseline RRi level at rest; *beta*, denotes the exercise-induced drop in the RRi; *c*, denotes the recovery proportion, relative to *beta*; *lambda* and *phi* denote the exercise-induced drop and recovery rate paramters; *tau* and *delta* denote the timing parameters, that controls when the drop occurs and the time duration of the exercise-induced depression on RRi.

| Parameter | Estimate | 95% CI | PD | PS | ESS | R-hat |
| --- | --- | --- | --- | --- | --- | --- |
| alpha Total lymphocytes | -0.03 | [-0.37, 0.31] | 0.582 | 0.350 | 9129.0 | 1.000 |
| alpha Total B lymphocytes | 0.20 | [-0.74, 1.21] | 0.660 | 0.581 | 6608.0 | 1.000 |
| alpha CD21 - CD11C + | 0.04 | [-0.32, 0.39] | 0.587 | 0.363 | 9664.9 | 1.000 |
| alpha CD21 - CD11C - | -0.31 | [-0.7, 0.09] | 0.935 | 0.847 | 9655.0 | 1.000 |
| alpha CD21 + CD11C - | -0.26 | [-1.1, 0.6] | 0.731 | 0.651 | 6980.6 | 1.000 |
| alpha CD21 + CD11C + | 0.48 | [0.11, 0.84] | 0.995 | 0.979 | 10444.5 | 1.000 |
| alpha sex [Male] | -0.36 | [-1.36, 0.63] | 0.752 | 0.692 | 6606.9 | 1.001 |
| alpha Age | 0.37 | [0.09, 0.63] | 0.997 | 0.977 | 9732.2 | 1.000 |
| alpha Body fat | -0.15 | [-0.48, 0.18] | 0.813 | 0.610 | 8603.7 | 1.000 |
| alpha Muscle mass | 0.03 | [-0.36, 0.46] | 0.552 | 0.362 | 7039.0 | 1.000 |
| beta Total lymphocytes | 0.17 | [-0.15, 0.53] | 0.845 | 0.661 | 8345.6 | 1.000 |
| beta Total B lymphocytes | 0.47 | [-0.5, 1.37] | 0.834 | 0.779 | 4782.0 | 1.000 |
| beta CD21 - CD11C + | -0.06 | [-0.41, 0.29] | 0.638 | 0.417 | 7269.2 | 1.000 |
| beta CD21 - CD11C - | -0.41 | [-0.8, -0.02] | 0.982 | 0.942 | 7957.0 | 1.000 |
| beta CD21 + CD11C - | -0.49 | [-1.3, 0.36] | 0.877 | 0.823 | 5150.7 | 1.000 |
| beta CD21 + CD11C + | 0.34 | [0, 0.71] | 0.973 | 0.915 | 8916.7 | 1.000 |
| beta sex [Male] | -0.54 | [-1.5, 0.44] | 0.860 | 0.809 | 5842.7 | 1.000 |
| beta Age | 0.28 | [0.02, 0.54] | 0.982 | 0.915 | 7852.7 | 1.000 |
| beta Body fat | -0.18 | [-0.5, 0.15] | 0.858 | 0.678 | 7587.0 | 1.000 |
| beta Muscle mass | 0.13 | [-0.26, 0.53] | 0.745 | 0.567 | 6461.4 | 1.000 |
| c Total lymphocytes | -0.27 | [-0.6, 0.06] | 0.950 | 0.847 | 10659.3 | 1.000 |
| c Total B lymphocytes | 0.09 | [-0.82, 1.03] | 0.576 | 0.495 | 6649.4 | 1.000 |
| c CD21 - CD11C + | -0.04 | [-0.37, 0.31] | 0.587 | 0.365 | 10543.0 | 1.001 |
| c CD21 - CD11C - | 0.34 | [-0.04, 0.72] | 0.961 | 0.892 | 11068.8 | 1.000 |
| c CD21 + CD11C - | 0.02 | [-0.79, 0.83] | 0.523 | 0.428 | 6705.4 | 1.000 |
| c CD21 + CD11C + | -0.24 | [-0.6, 0.1] | 0.915 | 0.791 | 12503.9 | 1.000 |
| c sex [Male] | -0.44 | [-1.4, 0.52] | 0.823 | 0.762 | 7719.4 | 1.000 |
| c Age | 0.05 | [-0.19, 0.32] | 0.668 | 0.360 | 11830.3 | 1.001 |
| c Body fat | 0.17 | [-0.14, 0.5] | 0.855 | 0.668 | 10016.1 | 1.000 |
| c Muscle mass | 0.04 | [-0.33, 0.44] | 0.579 | 0.380 | 8560.8 | 1.000 |
| lambda Total lymphocytes | 0.16 | [-0.19, 0.51] | 0.812 | 0.623 | 7625.7 | 1.000 |
| lambda Total B lymphocytes | -0.58 | [-1.59, 0.42] | 0.872 | 0.825 | 5037.1 | 1.000 |
| lambda CD21 - CD11C + | -0.24 | [-0.61, 0.13] | 0.902 | 0.768 | 6878.8 | 1.000 |
| lambda CD21 - CD11C - | 0.44 | [-0.01, 0.84] | 0.980 | 0.944 | 7525.2 | 1.000 |
| lambda CD21 + CD11C - | 0.34 | [-0.54, 1.21] | 0.783 | 0.709 | 5322.7 | 1.000 |
| lambda CD21 + CD11C + | -0.05 | [-0.43, 0.35] | 0.611 | 0.400 | 7911.5 | 1.000 |
| lambda sex [Male] | -0.21 | [-1.22, 0.82] | 0.658 | 0.584 | 5443.0 | 1.000 |
| lambda Age | 0.07 | [-0.2, 0.35] | 0.709 | 0.425 | 7926.5 | 1.000 |
| lambda Body fat | 0.09 | [-0.26, 0.44] | 0.699 | 0.478 | 7022.3 | 1.000 |
| lambda Muscle mass | 0.17 | [-0.27, 0.58] | 0.785 | 0.630 | 6609.5 | 1.000 |
| phi Total lymphocytes | 0.08 | [-0.28, 0.45] | 0.670 | 0.469 | 8309.0 | 1.000 |
| phi Total B lymphocytes | -0.51 | [-1.52, 0.51] | 0.836 | 0.782 | 6022.0 | 1.000 |
| phi CD21 - CD11C + | -0.10 | [-0.47, 0.28] | 0.704 | 0.508 | 7972.1 | 1.000 |
| phi CD21 - CD11C - | -0.20 | [-0.6, 0.25] | 0.822 | 0.675 | 8236.8 | 1.000 |
| phi CD21 + CD11C - | 0.59 | [-0.32, 1.46] | 0.899 | 0.858 | 6446.9 | 1.000 |
| phi CD21 + CD11C + | 0.23 | [-0.15, 0.62] | 0.876 | 0.746 | 9068.9 | 1.000 |
| phi sex [Male] | 0.55 | [-0.53, 1.57] | 0.855 | 0.805 | 6558.1 | 1.000 |
| phi Age | -0.13 | [-0.41, 0.14] | 0.825 | 0.593 | 8956.3 | 1.000 |
| phi Body fat | -0.02 | [-0.37, 0.33] | 0.552 | 0.331 | 7879.4 | 1.000 |
| phi Muscle mass | -0.19 | [-0.61, 0.25] | 0.800 | 0.652 | 7348.4 | 1.000 |
| tau Total lymphocytes | -0.19 | [-0.54, 0.16] | 0.870 | 0.713 | 10114.4 | 1.000 |
| tau Total B lymphocytes | -0.15 | [-1.15, 0.85] | 0.614 | 0.538 | 7109.7 | 1.000 |
| tau CD21 - CD11C + | 0.32 | [-0.04, 0.68] | 0.959 | 0.887 | 10093.2 | 1.000 |
| tau CD21 - CD11C - | -0.30 | [-0.68, 0.14] | 0.928 | 0.835 | 10568.8 | 1.000 |
| tau CD21 + CD11C - | 0.08 | [-0.84, 0.89] | 0.568 | 0.482 | 7503.5 | 1.000 |
| tau CD21 + CD11C + | 0.14 | [-0.22, 0.53] | 0.780 | 0.590 | 11440.1 | 1.000 |
| tau sex [Male] | -0.89 | [-1.83, 0.15] | 0.962 | 0.942 | 7418.1 | 1.000 |
| tau Age | 0.01 | [-0.27, 0.27] | 0.519 | 0.239 | 10772.1 | 1.000 |
| tau Body fat | -0.06 | [-0.39, 0.29] | 0.637 | 0.409 | 9775.4 | 1.000 |
| tau Muscle mass | 0.43 | [0.03, 0.84] | 0.979 | 0.945 | 8161.8 | 1.000 |
| delta Total lymphocytes | -0.16 | [-0.52, 0.2] | 0.804 | 0.621 | 8858.0 | 1.000 |
| delta Total B lymphocytes | -0.15 | [-1.21, 0.82] | 0.610 | 0.536 | 5981.7 | 1.000 |
| delta CD21 - CD11C + | 0.10 | [-0.27, 0.48] | 0.701 | 0.507 | 8881.6 | 1.000 |
| delta CD21 - CD11C - | -0.13 | [-0.53, 0.31] | 0.725 | 0.558 | 10292.3 | 1.000 |
| delta CD21 + CD11C - | 0.14 | [-0.73, 1.03] | 0.619 | 0.533 | 6164.3 | 1.000 |
| delta CD21 + CD11C + | 0.26 | [-0.12, 0.67] | 0.905 | 0.795 | 9793.2 | 1.000 |
| delta sex [Male] | -0.17 | [-1.22, 0.9] | 0.627 | 0.550 | 6067.9 | 1.000 |
| delta Age | -0.08 | [-0.36, 0.21] | 0.699 | 0.436 | 9402.4 | 1.001 |
| delta Body fat | -0.17 | [-0.53, 0.2] | 0.822 | 0.655 | 8754.7 | 1.000 |
| delta Muscle mass | -0.01 | [-0.43, 0.43] | 0.522 | 0.346 | 6796.0 | 1.000 |

Additionally, the residual correlations between model parameters from the adjusted model are illustrated in [Table S4](#tab-s4).

**Table S4**. Estimates of the residual correlation between model parameters controlling the exercise-induced cardiac autonomic response. Model effects are displayed adjusted by sex and age. *alpha*, denotes the baseline RRi level at rest; *beta*, denotes the exercise-induced drop in the RRi; *c*, denotes the recovery proportion, relative to *beta*; *lambda* and *phi* denote the exercise-induced drop and recovery rate paramters; *tau* and *delta* denote the timing parameters, that controls when the drop occurs and the time duration of the exercise-induced depression on RRi.

| Parameter | Estimate | 95% CI | PD | PS | ESS | R-hat |
| --- | --- | --- | --- | --- | --- | --- |
| alpha ~ beta | 0.24 | [0.03, 0.46] | 0.981 | 0.890 | 8967.0 | 1.000 |
| alpha ~ c | 0.02 | [-0.22, 0.25] | 0.576 | 0.258 | 11240.9 | 1.000 |
| beta ~ c | -0.18 | [-0.4, 0.04] | 0.935 | 0.756 | 10602.2 | 1.000 |
| alpha ~ lambda | 0.26 | [0.02, 0.46] | 0.983 | 0.907 | 9108.4 | 1.000 |
| beta ~ lambda | -0.36 | [-0.55, -0.15] | 0.999 | 0.990 | 9867.2 | 1.000 |
| c ~ lambda | 0.02 | [-0.21, 0.25] | 0.578 | 0.267 | 10447.9 | 1.000 |
| alpha ~ phi | 0.23 | [0.01, 0.46] | 0.973 | 0.871 | 9693.9 | 1.000 |
| beta ~ phi | -0.47 | [-0.64, -0.27] | 1.000 | 1.000 | 10616.7 | 1.001 |
| c ~ phi | -0.19 | [-0.41, 0.05] | 0.936 | 0.756 | 11180.8 | 1.000 |
| lambda ~ phi | 0.25 | [0.03, 0.46] | 0.983 | 0.908 | 10161.9 | 1.001 |
| alpha ~ tau | -0.12 | [-0.36, 0.1] | 0.846 | 0.576 | 12259.5 | 1.000 |
| beta ~ tau | 0.06 | [-0.18, 0.28] | 0.681 | 0.361 | 12213.6 | 1.000 |
| c ~ tau | 0.02 | [-0.23, 0.25] | 0.572 | 0.264 | 11227.2 | 1.000 |
| lambda ~ tau | -0.35 | [-0.55, -0.13] | 0.999 | 0.985 | 10196.5 | 1.000 |
| phi ~ tau | -0.08 | [-0.32, 0.15] | 0.741 | 0.430 | 10968.5 | 1.000 |
| alpha ~ delta | 0.07 | [-0.16, 0.3] | 0.711 | 0.392 | 10166.0 | 1.000 |
| beta ~ delta | -0.27 | [-0.48, -0.05] | 0.990 | 0.930 | 11070.1 | 1.000 |
| c ~ delta | -0.10 | [-0.33, 0.13] | 0.802 | 0.509 | 9576.8 | 1.000 |
| lambda ~ delta | 0.40 | [0.19, 0.59] | 0.999 | 0.996 | 10637.9 | 1.000 |
| phi ~ delta | 0.03 | [-0.2, 0.25] | 0.587 | 0.271 | 11185.3 | 1.000 |
| tau ~ delta | -0.19 | [-0.42, 0.04] | 0.942 | 0.782 | 9560.9 | 1.000 |

Moreover, the predicted cardiac autonomic kinetics to the full rest-exercise-recovery dynamics, adjusted by confounders, can be observed in [Figure S4](#fig-s4).


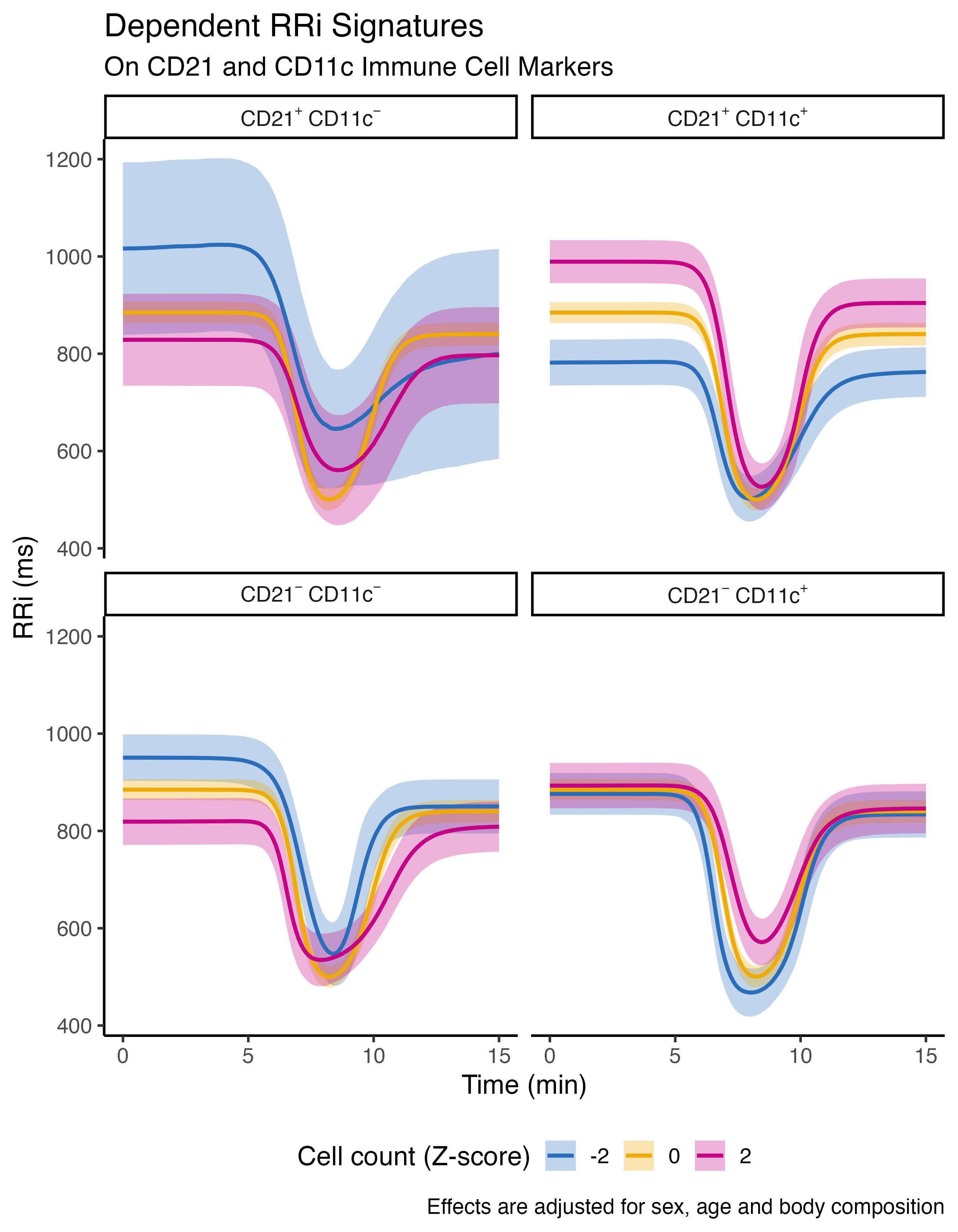
 **Figure S4**. Predicted cardiac autonomic signatures display different patterns dependent on the amounts of CD21 and CD11c B cell phenotypes (standardized units) and their corresponding cell subsets. These predicted dynamics are adjusted for sex and age.
